# Molecular determinants archetypical to the phylum Nematoda

**DOI:** 10.1186/1471-2164-10-114

**Published:** 2009-03-18

**Authors:** Yong Yin, John Martin, Sahar Abubucker, Zhengyuan Wang, Lucjan Wyrwicz, Leszek Rychlewski, James P McCarter, Richard K Wilson, Makedonka Mitreva

**Affiliations:** 1 The Genome Center, Department of Genetics, Washington University School of Medicine, St Louis, Missouri, USA; 2 Maria Sklodowska-Curie Memorial Cancer Center and Institute of Oncology, Warsaw, Poland; 3 Bioinfobank Institute, Poznan, Poland; 4 Divergence Inc, St Louis, Missouri, USA

## Abstract

**Background:**

Nematoda diverged from other animals between 600–1,200 million years ago and has become one of the most diverse animal phyla on earth. Most nematodes are free-living animals, but many are parasites of plants and animals including humans, posing major ecological and economical challenges around the world.

**Results:**

We investigated phylum-specific molecular characteristics in Nematoda by exploring over 214,000 polypeptides from 32 nematode species including 27 parasites. Over 50,000 nematode protein families were identified based on primary sequence, including ~10% with members from at least three different species. Nearly 1,600 of the multi-species families did not share homology to Pfam domains, including a total of 758 restricted to Nematoda. Majority of the 462 families that were conserved among both free-living and parasitic species contained members from multiple nematode clades, yet ~90% of the 296 parasite-specific families originated only from a single clade. Features of these protein families were revealed through extrapolation of essential functions from observed RNAi phenotypes in *C. elegans*, bioinformatics-based functional annotations, identification of distant homology based on protein folds, and prediction of expression at accessible nematode surfaces. In addition, we identified a group of nematode-restricted sequence features in energy-generating electron transfer complexes as potential targets for new chemicals with minimal or no toxicity to the host.

**Conclusion:**

This study identified and characterized the molecular determinants that help in defining the phylum Nematoda, and therefore improved our understanding of nematode protein evolution and provided novel insights for the development of next generation parasite control strategies.

## Background

The phylum Nematoda (roundworms) is one of the most common phyla of animals, estimated to contain over a million species [1]. Over 20,000 nematode species have been described [2], most of them are free-living but many are successful parasites of humans, animals, and plants, causing diseases of major socio-economic importance globally. Nearly three billion people are infected by the three most prevalent soil-transmitted intestinal worms, including roundworms (*Ascaris lumbricoides*), whipworms (*Trichuris trichiura*), and hookworms (*Necator americanus *and *Ancylostoma ceylanicum*) [3]. Tissue-dwelling filarial nematodes infect at least a billion people, causing river blindness (*Onchocerca volvulus*), elephantiasis (*Wuchereria bancrofti *and *Brugia malayi*), etc. In agriculture, the current financial losses caused by parasites to domesticated animals and crops greatly affect farm profitability and exacerbate challenges to global food production and distribution. For example, the root-knot nematodes *Meloidogyne spp*. and the cyst species (Globodera and Heterodera) cause an estimated $100 billion in annual damage [4].

Nematodes are believed to have diverged evolutionarily from other animals between 600–1,200 million years ago [2]. Proteins encoded by their genomes have experienced drastic changes since then, as evident in both expressed sequence tags (ESTs) [5-7] and genomes [8,9], and many are closely related to functional diversification, speciation, and species adaptation [10-14]. Among them are the nematode-specific proteins, which bear crucial importance for understanding nematode biology and parasitism [15-17]. In addition, studies on the proteins unique to nematodes can illustrate the roles of different genetic mechanisms, such as gene duplication and degeneration, retroposition, and *de novo *origination, in the emergence of novel proteins and protein families in nematodes. Furthermore, proteins that are specific to the pathogen or have sufficiently diverged from those in the host can be good targets for drugs with low toxicity to the host and the environment. Examples of such differential drug activities are antibiotics such as β-lactam and streptomycin and many anti-fungals [18].

Despite the importance of nematode-specific proteins and protein families, their representations are extremely limited in public databases. For example, 2,635 of the 8,296 protein families in Pfam-A [19,20] (v20) include nematode sequences, yet only 78 of them contain no members from non-nematode species and are thus putative nematode-specific families. This under-representation is a result of the quality control measures applied by the existing protein domain databases, such as Pfam, to restrict the sequences they incorporate to only the full length-proteins or those predicted from complete genomes [19]. Work by our laboratory and others have generated the vast majority of sequences currently available for many parasites from the phylum Nematoda as ESTs and genome survey sequences (GSSs) [21,22]. For example, transcriptomes of 38 nematode species, 32 of which are parasites of vertebrates or plants, have been sampled to generate over 510,000 ESTs [23]. However, putative nematode coding sequences among these ESTs and GSSs have never been explored systematically for the identification of nematode-specific protein-coding features.

To extend our and others' investigation of nematode evolution based on pan-phylum analyses [7,24,25], we have undertaken the challenge of identifying nematode-specific (or restricted) protein families using high-throughput computational methods developed to detect highly conserved coding regions in a robust fashion. From over 214,000 polypeptides in 32 nematode species including 27 parasites, we identified 758 protein families that were conserved in various nematode subgroups across the phylum Nematoda but were not represented in Pfam-A. These proteins were conserved in at least three species, therefore prospectively with essential functions, making them excellent candidates for the understanding of nematode evolution as well as targets for the broad control of nematodes. With cautions on the incompleteness of the currently available phylogenetic sampling, these nematode protein families were further categorized and characterized at functional and at structural levels. Most of them were conserved proteins with no functional annotations identified, a fraction of which were found to contain distant structural homology that may infer putative functions.

## Results and discussion

### Sequence organization

Sequence data is available for many nematode species primarily because of the recent sampling of nematode transcriptomes using ESTs [21,22]. In this study, a total of 130,357 contig-level EST consensus sequences, assembled from 262,497 ESTs from 29 nematode species, were translated into putative primary sequences of nematode proteins (Table 1). In addition, the complete gene-sets of 84,408 proteins from five genome sequencing projects (3 *Caenorhabditis *species, *B. malayi*, and *Ancylostoma caninum*) were added. Hence, a total of 214,159 polypeptides/proteins from 32 nematode species in four nematode clades were used for the subsequent analysis (Table 1). The complete dataset is available online for retrieval [26].

**Table 1:** Species and sequences.

Clades/Species		Code	# EST Contigs	# Poly-peptides
**EST contigs**				
V	*Ancylostoma caninum *^*a*^	AC	5,484	5,444
	*Ancylostoma ceylanicum *^*a*^	AE	4,953	4,954
	*Haemonchus contortus *^*a*^	HC	9,842	9,819
	*Nippostrongylus brasiliensis *^*a*^	NB	3,949	3,852
	*Ostertagia ostertagi *^*a*^	OS	4,831	4,821
	*Pristionchus pacificus *^*f*^	PP	2,654	2,654
IVa	*Parastrongyloides trichosuri *^*a*^	PT	4,934	4,925
	*Strongyloides ratti *^*a*^	SR	5,237	5,235
	*Strongyloides stercoralis *^*a*^	SS	3,479	3,478
IVb	*Globodera pallida *^*p*^	GP	2,973	2,960
	*Globodera rostochiensis *^*p*^	GR	2,530	2,528
	*Heterodera glycines *^*p*^	HG	2,026	2,016
	*Heterodera schachtii *^*p*^	HS	1,600	1,593
	*Meloidogyne arenaria *^*p*^	MA	3,372	3,354
	*Meloidogyne chitwoodi *^*p*^	MC	5,880	5,860
	*Meloidogyne hapla *^*p*^	MH	11,193	11,178
	*Meloidogyne incognita *^*p*^	MI	9,107	9,098
	*Meloidogyne javanica *^*p*^	MJ	5,172	5,162
	*Meloidogyne paranaensis *^*p*^	MP	2,263	2,252
	*Pratylenchus penetrant *^*p*^	PE	488	488
	*Radopholus similis *^*p*^	RS	788	789
	*Zeldia punctata *^*f*^	ZP	202	202
III	*Ascaris suum *^*a*^	AS	17,989	17,843
	*Brugia malayi *^*a*^	BM	1,609	1,517
	*Dirofilaria immitis *^*a*^	DI	2,534	2,527
	*Toxocara canis *^*a*^	TX	2,135	2,113
I	*Trichinella spiralis *^*a*^	TS	5,958	5,952
	*Trichuris vulpis *^*a*^	TV	1,690	1,681
	*Xiphinema index *^*p*^	XI	5,485	5,451
**Genes**				
V	*Ancylostoma caninum *^*a*^	AC		3,998
	*Caenorhabditis elegans *^*f*^	CE		23,162
	*Caenorhabditis briggsae *^*f*^	CB		19,723
	*Caenorhabditis remanei *^*f*^	CR		25,775
III	*Brugia malayi *^*a*^	BM		11,750

^a ^Animal parasite; ^b ^Plant parasite; ^f ^Free-living nematode.

### Building nematode protein families

**Figure 1:**
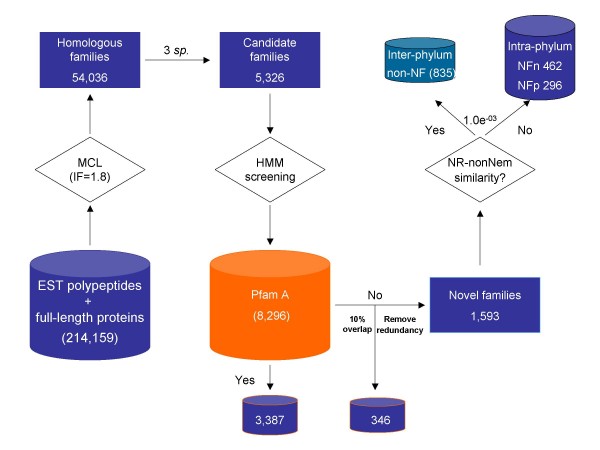
**Identification and classification of nematode-restricted protein families**.

Protein families were built using MCL clustering [27] with the Markov cluster algorithm (MCL), which would not suffer greatly from potential problems caused by multi-domain proteins, promiscuous domains, or fragmented sequences. In total, the 214,159 nematode coding sequences were clustered into 54,036 protein families. Protein families conserved across multiple species suggest conserved features and essential functions, therefore a total of 5,326 multi-species families (112,271 sequences), with members from at least three different nematode species, were chosen for further evaluation. Of these, 1,939 protein families (36%) did not share homology to protein family models in Pfam-A (Figure 1).

A protein family built by MCL can include multiple EST contigs originating from a single gene. To reduce this redundancy, we first clustered EST contigs into EST clusters, each containing a group of contigs likely representing the same gene [28-31]. Then a step-wise approach was implemented for each MCL family to: i) generate a multiple alignment from all members, ii) build a Hidden Markov model (HMM) from the multiple alignment, iii) calculate a matching score for each member of the family based on the HMM, and iv) retain only the single EST contig from each EST cluster assigned with the best matching score as the sole representative of the gene in the protein family. Finally, an additional filtering step required each valid family to have at least 10% (in length) of its full alignment contributed simultaneously by sequences from 3 or more species. All of the above led to the identification of 1,593 multi-species non-Pfam Nematode protein Families (NFs) with a total of 13,963 coding sequences (Figure 1).

### Identification of novel phyla-restricted nematode protein families

The NFs were further categorized by sequence similarities and taxonomic origins of their members. Comparison to the NR-noNema database (all protein sequences in the non-redundant NR database except those from nematodes), at a BLAST *e*-value cutoff of 1.0e^-03^, identified 835 NFs (8,764 proteins) containing homology in non-nematode species although they were derived from nematodes (Figure 1) (see Additional file 1). Approximately 90% of these NFs shared primary sequence similarities to arthropod proteins, among which 26 families (NFa; 212 sequences) were found to be homologous only to sequences from arthropods but not to any proteins from non-nematode and non-arthropod species at a BLAST *e*-value cutoff of 1.0e^-03 ^(see Additional file 2). Molecular features conserved in the sequences of both nematodes and arthropods were evident in these families, such as small insertions/deletions [see Additional file 3]. Both Nematoda and Arthropoda belong to Ecdysozoa, sharing the common pattern of growth-by-molting [32,33], therefore these protein families likely reflect the evolutionary conservation between these organisms at the molecular level. In addition, macrocyclic lactones, such as avermection and milbemycin, have been successfully used as endectocides to treat both the nematode endoparasites and arthropod ectoparasites simultaneously [34]. Hence, the 26 NFs that were conserved only among nematodes and arthropods could be potential targets for the development of novel endectocides. Interestingly, five of the 26 families were mapped to canonical KEGG metabolic enzymes [35-37] as various subunits of the electron transfer Complex I [see Additional file 2].

The remaining 758 NF families (5,199 sequences) did not contain members with sequence similarities to any non-nematode proteins with the BLAST *e*-value cutoff of 1.0e^-03 ^(Figure 1). With no obvious homology to either non-nematode proteins or Pfam-A entries, they became candidate novel protein families specific (or restricted) to nematodes. Their conservation among multiple nematode species, especially of those spanning all the four nematode clades (Table 2) (phylogeny based on [38]) included in this study (see below), suggests that they may have emerged in early nematode ancestors after they diverged from other animals, and they may include the molecular determinants archetypical to the phylum Nematoda. Although their nematode-specificity implies only limited knowledge currently available, close investigation will likely reveal conserved functions essential to many nematodes, and the interference with their functions will likely cause severe damaging effects in nematode parasites in a novel, safe, and broad fashion.

**Table 2:** NF families spanning the four clades.

NFs	Members (#)	SP	TM	Struct. Homology	Intfam	KEGG Annotation	InterPro Mapping	RNAi
**NFp**								
NF_0405_1573	5	+	+	-		-	IPR013032 (EGF-like region)	-
NF_0410_0798	13	-	+	-		-	-	-
**NFn**								
NF_0404_1399	5	-	-	-	-	-	-	-
NF_0406_0090	6	-	+	-	-	-	-	Dpy Let unc Prl transgene_expression_increased Gro
NF_0407_1004	8	-	+	-	-	-	-	fat_content_increased
NF_0407_1250	7	-	+	-	-	-	-	WT
NF_0407_1301	8	+	-	-	-	-	-	transgene_expression_increased WT
NF_0408_0068	8	+	+	-	-	-	IPR000583 (Glutamine amidotransferase, class-II)	Ric
NF_0408_0121	11	+	+	-	-	-	-	unc thin Lon Gro WT
NF_0408_0187	12	+	-	+	-	-	-	WT
NF_0408_0355	8	+	+	-	-	-	-	WT
NF_0408_0750	8	+	+	-	-	-	-	WT
NF_0408_1462	9	+	+	-	-	-	IPR002057 (Isopenicillin N synthetase)	-
NF_0409_1025	11	+	+	-	-	-	-	WT
NF_0410_0459	14	+	-	-	-	-	IPR010345 (Interleukin-17) IPR000173 (Glyceraldehyde 3-phosphate dehydrogenase)	WT
NF_0412_0004	12	-	+	-	-	-	-	WT
NF_0412_0519	13	+	-	-	-	-	-	unc Prl unclassified Rup transgene_localization_abnormal Gro
NF_0412_0625	13	-	-	-	-	-	IPR005374 (Protein of unknown function UPF0184) IPR009053 (Prefoldin)	Clr unc fat_content_reduced Gro
NF_0412_1508	12	+	+	-	-	-	-	unc Lva Gro
NF_0412_1534	14	+	-	-	-	-	-	WT
NF_0413_0363	14	-	+	-	-	-	-	Bmd Let Lva Emb reduced_brood_size
NF_0413_1248	14	+	+	-	-	-	IPR008263 (Glycoside hydrolase, family 16, active site)	unc
NF_0414_0910	30	+	+	-	-	-	IPR014756 (Immunoglobulin E-set)	WT
NF_0416_0115	21	-	-	-	-	-	-	WT
NF_0417_1395	19	-	-	-	+	-	-	Muv
NF_0419_1162	19	-	+	-	+	K03960 (NADH dehydrogenase (ubiquinone) 1 beta subcomplex 4)	IPR009866 (NADH-ubiquinone oxidoreductase, subunit NDUFB4)	Bmd Lva Emb
NF_0423_0313	35	+	+	-	-	-	IPR013032 (EGF-like region)	WT

**Figure 2:**
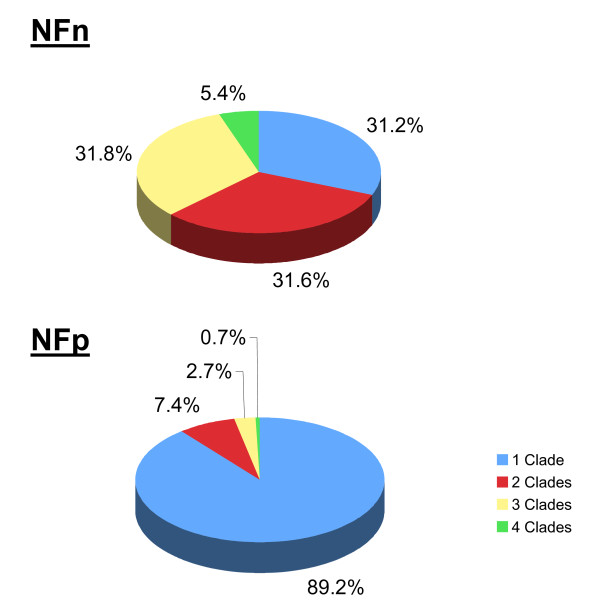
**Phylogenetic distribution by members of the NFn and NFp families**. A similar number of NFn families were found to span 1, 2, or 3 nematode clades respectively (~31% for each). In contrast, the majority in the NFp group (90%) contained members from only a single nematode clade.

In addition, by comparing to a database containing all the currently available sequences from free-living nematodes (at a BLAST *e*-value cutoff of 1.0e^-05^), these nematode-restricted NFs were further divided into 296 NFp (putatively specific to parasitic nematodes) [see Additional file 4] and 462 NFn (conserved across both free-living and parasitic nematodes) [see Additional file 5]. The NFp and NFn groups contained 1,514 and 3,685 proteins, respectively, averaging at 5 and 8 members per family with different family size distributions [see Additional files 1 and 6], suggesting differences between the two groups. In addition, while the majority in the NFp group (90%) contained members from only a single nematode clade, a similar number of NFn families were found to span 1, 2, or 3 nematode clades respectively (~31% of all NFn for each) (Figure 2). These results indicate that proteins within the NFn families are conserved in more evolutionarily divergent nematode species and are thus likely involved in essential nematode function across the phylum Nematoda; on the other hand, the NFp families tend to be restricted to smaller evolutionary niches and are most likely related to the specific patterns of parasitism that were hypothesized to emerge independently, at multiple times, during nematode evolution [38].

### NF families containing *C. elegans *members with RNAi phenotypes

RNA interference (RNAi) has become an efficient high-throughput approach for rapidly determining gene functions via transcript knockdown in many organisms, and especially in *C. elegans *[39-42]. However, applying RNAi in parasitic nematodes possesses significant challenges. For example, their obligate parasitic life cycles, with movement into and out of the host, make both the delivery of double-stranded RNA and the assessment of phenotype difficult. Although successes have been demonstrated in several parasitic nematode species (reviewed in [21,43]), these methodologies are far from established for large-scale investigation.

Gene functions derived from RNAi experiments in *C. elegans *can be further extrapolated, to an extent, to orthologous genes in other nematodes [21]. The NFp families did not have members from the free-living *C. elegans*. A total of 356 of the 462 NFn families had *C. elegans *members, most of them (321) had RNAi results available. Among them, 85 families contained *C. elegans *genes associated with non-wild type RNAi phenotypes, including 62 with strongly deleterious effects (Emb, Ste, Stp, Lva, Lvl, and Gro) [see Additional file 5]. Such RNAi results could shed light on the putative functions of their counterparts in other nematodes included in the same protein families. For example, NF_0208_1522 contained two members from each of the three clade V free-living *Caenorhabditis *species, as well as four from animal parasites (AC02485 and OS00413 from clade V and SS02646 and PT01276 from clade IVa), and one from the clade IVb plant parasite *M. incognita *(MI03217). The inclusion of the two *C. elegans *insulin-like genes, ins-17 and ins-18, suggested that this family represented a group of conserved nematode proteins likely regulating the growth and lifespan as demonstrated by RNAi in *C. elegans*.

**Figure 3:**
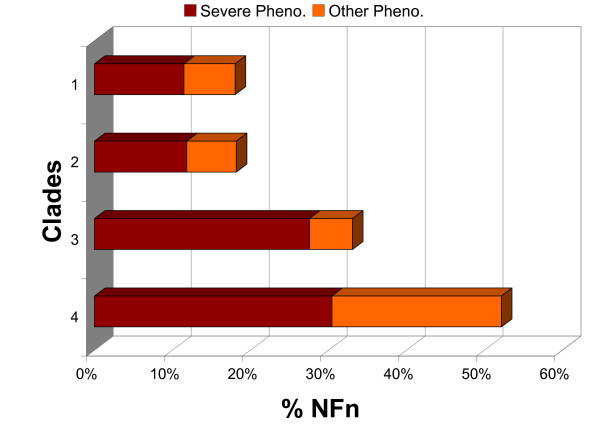
**RNAi phenotypes of *C. elegans *members in the NFn families**. Families conserved in nematodes spanning a broader evolutionary distance, especially those with members from all the four nematode clades included in this study, were much more likely to have observable phenotypes with RNAi knockdown in *C. elegans*. Severe Pheno., strongly deleterious effects including Emb, Ste, Stp, Lva, Lvl, and Gro; Other Pheno., other observable phenotypes.

Furthermore, the distribution of these RNAi results among NFn families showed that families conserved in nematodes spanning a broader evolutionary distance, especially those with members from all the four nematode clades included in this study, were much more likely to have observable phenotypes with RNAi knockdown in *C. elegans *(Figure 3). This suggested that these multi-clade NFn families, which might have emerged in the early common ancestors of Nematoda and remained to be conserved in many nematode species since then, could be the most essential genes required for nematode survival.

### NF families with functional annotations

Based on sequence similarities, members of the NFs were mapped to the Kyoto Encyclopedia of Genes and Genomes (KEGG), which offers curated information about genes and proteins, as well as molecular wirings of interactions and reaction networks especially in the canonical metabolic pathways [36,37]. As expected for these novel families, none of the NFp members could be mapped, and the limited assignments for the NFn families were always derived from their *C. elegans *members that were previously annotated by KEGG [see Additional file 5]. In addition, for each of the eight NFn families mapped through KEGG, a same KEGG Orthology (KO) entry was always assigned consistently to all of its family members meeting the mapping criteria (Table 3), confirming that the family members grouped by MCL were indeed homologous proteins.

**Table 3:** KEGG mappings for NFn families.

NFn Families	# Members	# Mapped	EC Enzyme	KEGG Orthology	KEGG Pathway
NF_0103_0353	3	1	4.2.1.1	E4.2.1.1: carbonic anhydrase (K01672)	Nitrogen metabolism (ko00910)
NF_0203_0963	5	2	-	Potassium channel, subfamily K, invertebrate (K05323)	
NF_0207_1379	9	8	1.6.5.3 1.6.99.3	NDUFS5: NADH dehydrogenase (ubiquinone) Fe-S protein 5 (K03938)	Oxidative phosphorylation (ko00190)
NF_0308_0938	12	9	1.6.5.3	ND4L: NADH dehydrogenase I subunit 4L (K03882)	Oxidative phosphorylation (ko00190)
NF_0312_1355	13	7	1.10.2.2	QCR10: ubiquinol-cytochrome c reductase subunit 10 (K0420)	Oxidative phosphorylation (ko00190)
NF_0313_0956	13	11	1.6.5.3 1.6.99.3	NDUFA7: NADH dehydrogenase (ubiquinone) 1 alpha subcomplex 7 (K03951)	Oxidative phosphorylation (ko00190)
NF_0320_0609	30	25	1.9.3.1	COX6C: cytochrome c oxidase subunit Vic (K02268)	Oxidative phosphorylation (ko00190)
NF_0419_1162	19	19	1.6.5.3 1.6.99.3	NDUFB4: NADH dehydrogenase (ubiquinone) 1 beta subcomplex 4 (K03960)	Oxidative phosphorylation (ko00190)

Unexpectedly, all the eight KEGG entries assigned to NFn proteins, such as the various subunits of electron-transfer complexes (Table 3), were canonical enzymes with extensive knowledge available, including sequences of orthologous groups from many non-nematode species. It was intriguing because the proteins included in these nematode families, especially the *C. elegans *members that had been previously annotated in KEGG, had to contain a fair amount of sequence homology to be recognized as the canonical enzymes, yet they were found without similarities to any non-nematode proteins by our discovery pipeline. Close examination showed that this conflict was caused by a slightly looser requirement of homology during the KEGG mapping. Therefore, the putative annotation assigned to these nematode proteins represented the relatively low levels of sequence similarities that were still able to reveal their functions with confidence.

**Figure 4:**
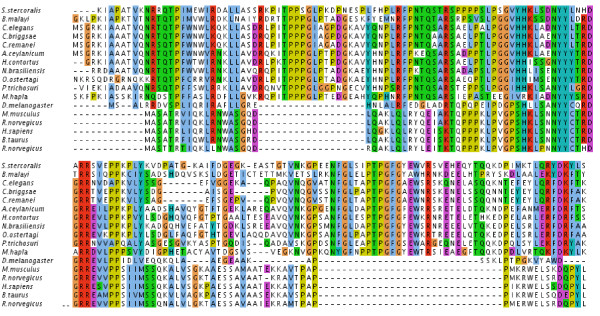
**Nematode-specific sequence features in the NF families**. Insertions specific to nematodes were evident in the global multiple alignment among members of NF_0313_0956 and orthologous proteins from non-nematode species. NF_0313_0956 included SS00822 (*Strongyloides stercoralis*), 14968.m01483 (*Brugia malayi*), F45H10.3 (*Caenorhabditis elegans*), gi-39591288-emb-CAE73341.1-(*Caenorhabditis briggsae*), cr01.Contig9.wum.334.1 (*Caenorhabditis remanei*), AE04133 (*Ancylostoma ceylanicum*), HC05738 (*Haemonchus contortus*), NB03814 (*Nippostrongylus brasiliensis*), OS04039 (*Ostertagia ostertagi*), PT04092 (*Parastrongyloides trichosuri*), and MH00982 (*Meloidogyne hapla*). Non-nematode orthologous proteins were those annotated as the NADH dehydrogenase (ubiquinone) 1 alpha subcomplex 7 (KO: K03951) from fly, bovine, mouse, rat, and human.

More interestingly, we were able to identify unique sequence features of these nematode proteins, such as nematode-specific insertions and deletions, in all the eight NFn families with KEGG annotations. Such nematode-specific features may have prevented their homology from being identified in our initial screening. For example, members of NFn family NF_0313_0956 were mapped to KO: K03951 as the NADH dehydrogenase (ubiquinone) 1 alpha subcomplex 7. Indeed, these nematode sequences could be forcibly aligned with the group of proteins from non-nematode organisms that were assigned to the same KEGG entry, after allowing two fragments of nematode-specific insertions (Figure 4). The lack of a homologous 3D model of this enzyme made it impossible to investigate the impact on its structure caused by these insertions, but they likely created additional loops in the nematode proteins that may introduce novel functional features specific to Nematoda. These results demonstrated the mechanism of directed diversification of existing protein folds in these proteins during nematode evolution.

### Energy generation in nematodes

Energy generation mechanisms are extremely complicated in nematodes. Free-living nematodes, such as *C. elegans*, rely on mammalian-type aerobic electron transfer for the generation of ATP. However, this oxygen-based energy generation mechanism is thought to be unlikely for many parasites because of the low levels of oxygen in their parasitic environments and the lack of an efficient circulatory system and respiratory organs in nematodes. Instead, an anaerobic energy generation independent of oxygen has been suggested. Studies of the clade III intestinal parasite *Ascaris suum *have revealed that a developmental switch around stage L3, wherein an anaerobic pathway in adults, named the malate dismutation pathway or the PEPCK-succinate pathway, replaces the mammalian-type aerobic energy generation found in embryos and larvae [44-47]. Our previous investigation of the adult transcriptome from another clade III parasite *Dirofilaria immitis *has suggested a similar mechanism [28].

With KEGG mapping, we identified a total of six components of the well-defined energy-generating electron transfer complexes among NFn families, each with relatively weak yet clear homology to the canonical enzymes. Based on this, and the finding that five NFa families conserved in only nematodes and arthropods were also mapped to the same pathway [see Additional file 4], we propose that the early common ancestors of nematodes may have obtained a series of novel features in their energy generation to collectively and cooperatively accommodate the severe challenges imposed by the different life styles found in complex parasitism, and that those NFa families may have represented an intermediate evolutionary path, which would have emerged in the common ancestors of Ecdysozoa, that leads to unique features specific to Nematoda. This phyla-specific energy generation mechanism, significantly distinct from the canonical pathway of oxidative phosphorylation used by mammalian hosts, offers a prime target for the development of next generation parasite control strategies with potentially high specificity and minimal toxicity.

### NF families with distant sequence homology

To offer further characterization, NF families were scanned against the InterPro database [48] for generic sequence features [see Additional files 4 and 5]. Not surprisingly, even with both KEGG and InterPro mappings, we were able to obtain information for only 30 NFp and 124 NFn families, leaving the majority of the NF families (~80%, 606/758) completely un-annotated.

Protein structure diverges more slowly than primary sequence [49], therefore fold similarity and structure-based alignments were used for the detection of distant homology of the 606 NF families with no KEGG or InterPro annotation. Firstly, we generated predictions of basic structural information for each, including secondary structure, domain architecture, and flexible/dynamic regions. These predictions are integrated and displayed within a customized genome browser [50] for easy navigation [26]. Secondly, structural homology to previously defined protein folds in Protein Data Bank (PDB) [51] or Pfam [19,20] were searched for using a newly improved version of the meta-predictor, Meta-BASIC [52], which combines sequence profile, secondary structure, and prediction of the burial states of individual amino acid with various scoring systems and meta profile alignment algorithms. The putative matches with a confident 3D-Jury cutoff score of 50, which corresponds to a false positive rate of less than 5% [49], are available online via graphic display [26]. Of the 3,926 sequences from the 606 families, we were able to identify putative homology for 56 polypeptides from 9 families to known protein folds in PDB, and 14 in 11 families to those in Pfam [see Additional files 4 and 5]. Close investigation of such distant homology can help to elucidate potential function (as described below).

**Figure 5:**
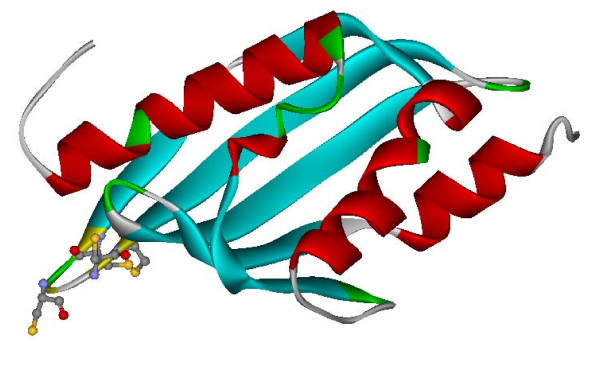
**Structural simulation of a conserved domain in NF_0103_0974**. The structure of a domain of 136 amino acids, conserved in all the five members of NF_0103_0974, were computationally simulated based on its distant homology to the PDB entry 1buqa. All the major components of 1buqa were preserved in this nematode domain, and the presence of a cysteine cluster might suggest a zinc-binding site.

An example is family NF_0103_0974 with a domain of 136 amino acids conserved in all the five members. Several structure prediction methods included in Meta-BASIC, such as the homology modeling tool FFAS3 [53] and threading algorithms 3D-PSSM [54] and INUB [55], all assigned this conserved domain as a match to the PDB entry 1buqa, which was classified as the structure signature for a group of nuclear transport factor 2 (NTF2) like proteins. Further structural modeling using Modeller [56] showed that the nematode domain contained all the major components of this fold. The NTF2-like superfamily contains members with diverse functions, including enzymes such as enscytalone dehydratase, delta-5-3-ketosteroid isomerase, and limonene-1,2-epoxide hydrolase, and non-enzymatic homologues such as NTF2 [57]. Even though none of these functions could be clearly assigned to NF_0103_0974, the presence of a cysteine cluster might suggest the existence of zinc binding site in these nematode proteins (Figure 5).

### NF families on accessible surfaces

Proteins secreted or expressed at surfaces are essential components of the cellular regulatory networks that ensure proper interactions with the environment for survival. Thus far, all the commercially available anthelmintics have a gain-of-function mode of action targeting channels and receptors associated with membranes [58]. In addition, nematode antigens are believed to be most effective when secreted from glands [59] or expressed on exposed surfaces such as the intestinal lumen in hookworm [60], where they come into contact with and are therefore targeted by effector molecules from the host immune system. Among the NFn and NFp families, there were 45% and 27%, respectively, having signal peptide for secretion predicted in their sequences, and 26% and 21%, respectively, containing members predicted to have both signal peptide and transmembrane domains. With the caution that some of these predictions might be putative targeting signals for transport to intracellular compartments such as mitochondria or peroxisomes, we were able to identify 149 NF families, from the total of 758, as candidates for expression at accessible surfaces [see Additional files 4 and 5].

The intestine has been our focus in other studies [61], because it is one of the major organs in nematodes creating a key surface at the intestinal apical membrane to interact with the environment. The easy accessibility of the nematode intestine has made it an attractive target for immune or chemical control of parasitic species [62-67]. Comparative studies among intestinal transcriptomes from the free-living *C. elegans *and parasites *A. suum *and *H. contortus *identified a group of 241 protein families (IntFam-241) expressed in the intestine of all three nematodes. This group was further proposed to represent an ancient group of intestinal proteins responsible for the core intestinal functions in many nematode species [61]. There were 12 NFn families from this study overlapping with the IntFam-241. Majority of them (11/12) spanned three or more nematode clades, and eleven had predictions of either signal peptides or transmembrane sequences [see Additional files 4 and 5]. In addition, all of the 12 NFn families had *C. elegans *members with RNAi information available, and all but two of them had observable RNAi phenotypes [see Additional file 5 and Additional file 6], suggesting that they warrant further investigation.

## Conclusion

Genomics studies of parasites from the phylum Nematoda have been mainly restricted to EST-based surveys of transcriptomes [23]. Beyond *C. elegans*, more than 520,000 ESTs have been generated from more than 40 species. As next-generation sequencing technologies drive cost down significantly, the sequencing of complete genomes of many eukaryotic species, including parasitic nematodes, can be foreseen in the near future. Nematologists currently have genome sequences available from nine nematode species including three parasites. The first annotated genome of a parasitic nematode, *Brugia malayi*, contained over 11,000 genes [68]. Recently the genome of plant parasite *Meloidogyne incognita *became available with over 19,000 genes [69]. New anti-parasitic drug targets were identified through investigations of both genomes. The human parasite *T. spiralis *is a significant food safety concern and an evolutionary out-group to many other nematodes [70]. The annotation of its genome has been completed and extensive comparative studies are currently underway (Mitreva, unpublished). In the next five years, collaborative projects at the Genome Center at Washington University and the Wellcome Trust Sanger Institute will increase the available parasitic nematode sequences by another order of magnitude, adding a total of 25 draft genomes supplemented by numerous cDNA reads with pyrosequencing. However, we anticipate that their complete annotated genomes are still 2–4 years away. Until then, transcriptomic data will remain the main source of information for the investigation of nematodes at the molecular level.

Currently, the primary control of parasitic nematode infection is based upon chemical treatments (anthelmintics). However, the incomplete protective response of the host and the acquisition of anthelmintic resistance by an increasing number of parasitic nematodes have hampered what used to be effective control strategies. Moreover, the use of drugs poses the risk of residue problems in meat, milk, and the environment. With minor exceptions, vaccines do not exist against parasitic nematodes of mammals, although immunity can develop against many of these pathogens. Hence, better understanding of the unique molecular characteristics in nematodes and a way of target prioritization is essential.

The pan-phylum analyses presented here demonstrate how genomics-based methods can offer a growing and fundamental information base, which, when coupled with extensive functional and structural annotations, can improve our understanding of the protein evolution in the phylum Nematoda through identification and characterization of the unique molecular features, and provide useful information in the identification and characterization of target proteins for the development of vaccines and next-generation anthelmintic drugs with a broad application.

## Methods

### Partial and complete genomes

Detailed information on genetic materials and cDNA library construction are available online [23,26]. ESTs were processed and clustered as described earlier [28-31]. EST contig sequences were translated individually by Prot4EST, a 6-tier translation pipeline combining both similarity-based methods and *de novo *predictions [71,72]. Only one translation was accepted to represent each EST contig, during which false translation was likely reduced by retaining preferably the longest open reading frame with strong supporting evidence, if available, in the form of similarities to known or predicted proteins. The gene-sets from the genomic sequencing projects were: *C. elegans *(Wormbase v158; 23,162 proteins), *C. briggsae *(downloaded June, 2006; 19,723 proteins), *C. remanei *(preliminary set, October, 2005; 25,775 proteins), *B. malayi *(11,750 proteins), and *A. caninum *(preliminary set; 4,038 proteins).

### Sequence comparison

WU-BLASTP (wordmask = seg postsw) was used to query the translated sequences against protein databases, and WU-TBLASTN (wordmask = seg lcmask) for searching against nucleotide databases [73]. Databases used for sequence comparisons were: i) NR-noNema, containing all sequences from the non-redundant protein database NR except those from nematodes (downloaded 06/06/2007), ii) NR-noNema-noArthropoda, NR sequences with those originated from nematode and arthropod species removed (downloaded 06/06/2007), iii) Free-living, all the 71,496 protein sequences from the free living species *C. elegans*, *C. briggsae*, *C. remanei*, *P. pacificus*, and *Z. punctata*.

### Building nematode protein families using MCL clustering

An all-against-all WU-BLASTP was performed on the total of 214,159 translated sequences from the 32 nematode species. Raw BLAST results were fed to a C-language implementation of Markov cluster algorithm [74], a fast and scalable unsupervised clustering algorithm based on simulation of flow in graphs [27]. MCL simulates flow in a protein similarity graph, assigning complete protein sequences into families based on density and strength between them. It makes no attempt to decompose the sequences into their component domains, but rather produces protein clusters that correlate well with the overall domain architecture. The tightness of MCL clustering is determined by a user-defined parameter, the inflation factor. A larger inflation factor leads to a higher granularity of clustering, resulting in the generation of protein families based more likely on local domains and less likely on global similarities. To avoid such high granularity, we validated the clustering with three inflation factors, 1.6, 1.8, and 2.0, respectively. With them, the numbers of protein families differed by only 5% (data not shown). After manual inspection, the inflation factor of 1.8 was chosen to generate 54,036 protein families for further screening and downstream analysis. HMM screening against Pfam-A entries (v20; 8,296 entries) [19,20] were performed using hmmpfam in HMMER v2.1 at a *p*-value cut-off 1.0e^-05^. To remove sequence redundancy, an automatic screening pipeline was implemented to: i) generate multiple alignments for each family with MUSCLE v3.52 [75], ii) build a HMM from the multiple alignment using hmmbuild from HMMER v2.1, iii) calculate a matching score for each member of the family based on the HMM using hmmsearch from HMMER v2.1, and iv) retain only one EST contig assigned with the best matching score for an EST cluster, each of which can be approximated as the collection of EST contigs originated from a single genomic locus.

### Prediction of signal peptide and transmembrane domain

A hidden Markov modeling-based algorithm, Phobius [76], was used with default settings. Each query sequence was further annotated as SP for containing signal peptide, TM for containing transmembrane region, or intracellular, based on raw Phobius outputs.

### KEGG and InterPro mappings

The *E*-value cut-off of 1.0e^-10 ^reported by WU-BLASTP against the Genes Database Release 43.0 from Kyoto Encyclopedia of Genes and Genomes (KEGG) was used for pathway mappings. For each query, the top match and all the matches within a range of 30% of the top BLAST score, if meeting the cut-off, were accepted for valid KEGG associations [35-37]. Default parameters for InterProScan v16.1 [77] were used to search against the InterPro database [48].

### Protein structural analysis

The following structural information was predicted for the NF family members: secondary structure prediction by PsiPred [78], domain architecture with SSEP-Domain [79], detection of flexible and dynamic regions using DISOPRED2 [80]. To investigate distant homology, query sequences were submitted to Meta-BASIC [52] against data sets of meta-profiles derived from PDB [51] and Pfam [19,20]. For the proteins identified as putative matches by Meta-BABIC, potential globular regions were identified with GlobPlot [81], and were subsequently submitted to the Structure Prediction Meta Server [82] for additional analyses. Secondary structure prediction was performed with PsiPred and ProfSec *via *the meta server, collected models were screened with 3D-Jury [83], a consensus fold recognition prediction method, for final predictions. Homology models were further obtained with Modeller version 6.2 [56] with additional refinement of structural alignments performed according to the Verify3D results as previously described [84].

## Abbreviations

EST: expressed sequence tag; GSS: genome survey sequence; HMM: Hidden Markov model; KEGG: the Kyoto Encyclopedia of Genes and Genomes; KO: KEGG orthology; MCL: Markov cluster algorithm; NF: nematode protein family; NFn: NF families conserved across both free-living and parasitic nematodes; NFp: NF families putatively specific to parasitic nematodes; PDB: Protein Data Bank; RNAi: RNA interference.

## Authors' contributions

YY, JPM, RKW, and MM conceived of the study. YY, JM, SA, ZW, LW, and LR carried out analyses. YY and MM interpreted results and prepared the manuscript. All authors read and approved the final manuscript.

## Supplementary Material

Additional file 1 **Statistics of the NemFam groups. **This table lists the comparison of statistics by different protein family groups. Click here for file

Additional file 2 **Annotation of the NFa groups. **This table includes details of the NFa groups, including functional and structural annotations. Click here for file

Additional file 3 **Multiple alignment among members of the NFa family NF_0308_1018. **The members of NF_0308_1018 were aligned with the orthologous proteins annotated to KO:K0938 as NADH dehydrogenase (ubiquinone) Fe-S protein 5. Click here for file

Additional file 4 **Annotation of the NFp groups. **This table includes details of the NFp groups, including functional and structural annotations. Click here for file

Additional file 5 **Annotation of the NFn groups. **This table includes details of the NFn groups, including functional and structural annotations. Click here for file

Additional file 6 **Size distribution of NFn and NFp groups. ** This figure shows the different distribution of family sizes by the NFn and NFp groups. Click here for file
