# Supplementary figures and images for: Molecular determinants archetypical to the phylum Nematoda
Source: BMC Genomics. 2009 Mar 18;10:114. doi: 10.1186/1471-2164-10-114 (PMC2666764; doi:10.1186/1471-2164-10-114)

## Slide 1
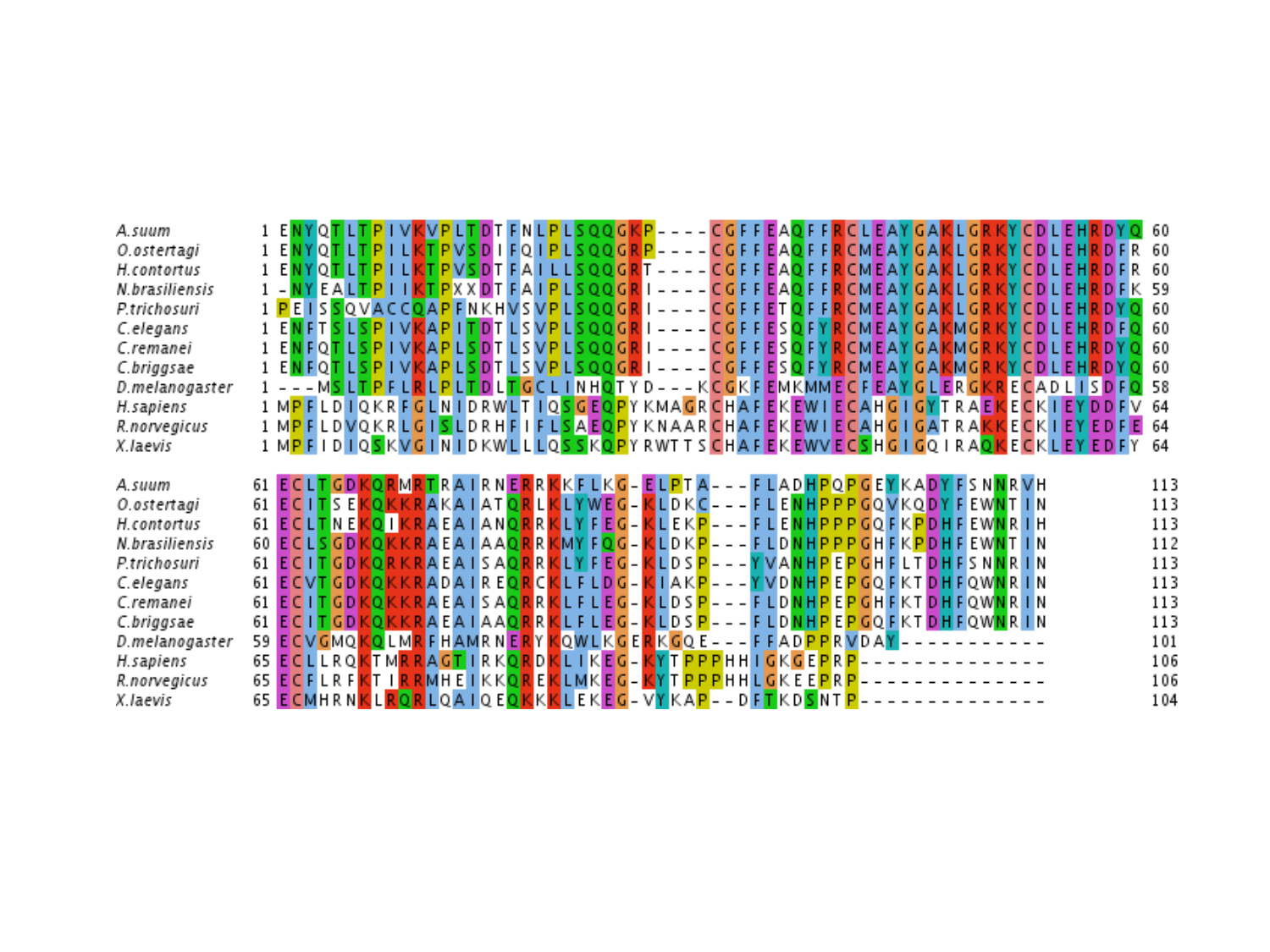

Supplement: Additional file 3 — Multiple alignment among members of the NFa family NF_0308_1018. The members of NF_0308_1018 were aligned with the orthologous proteins annotated to KO:K0938 as NADH dehydrogenase (ubiquinone) Fe-S protein 5. [file 1471-2164-10-114-S3.ppt]

## Slide 1
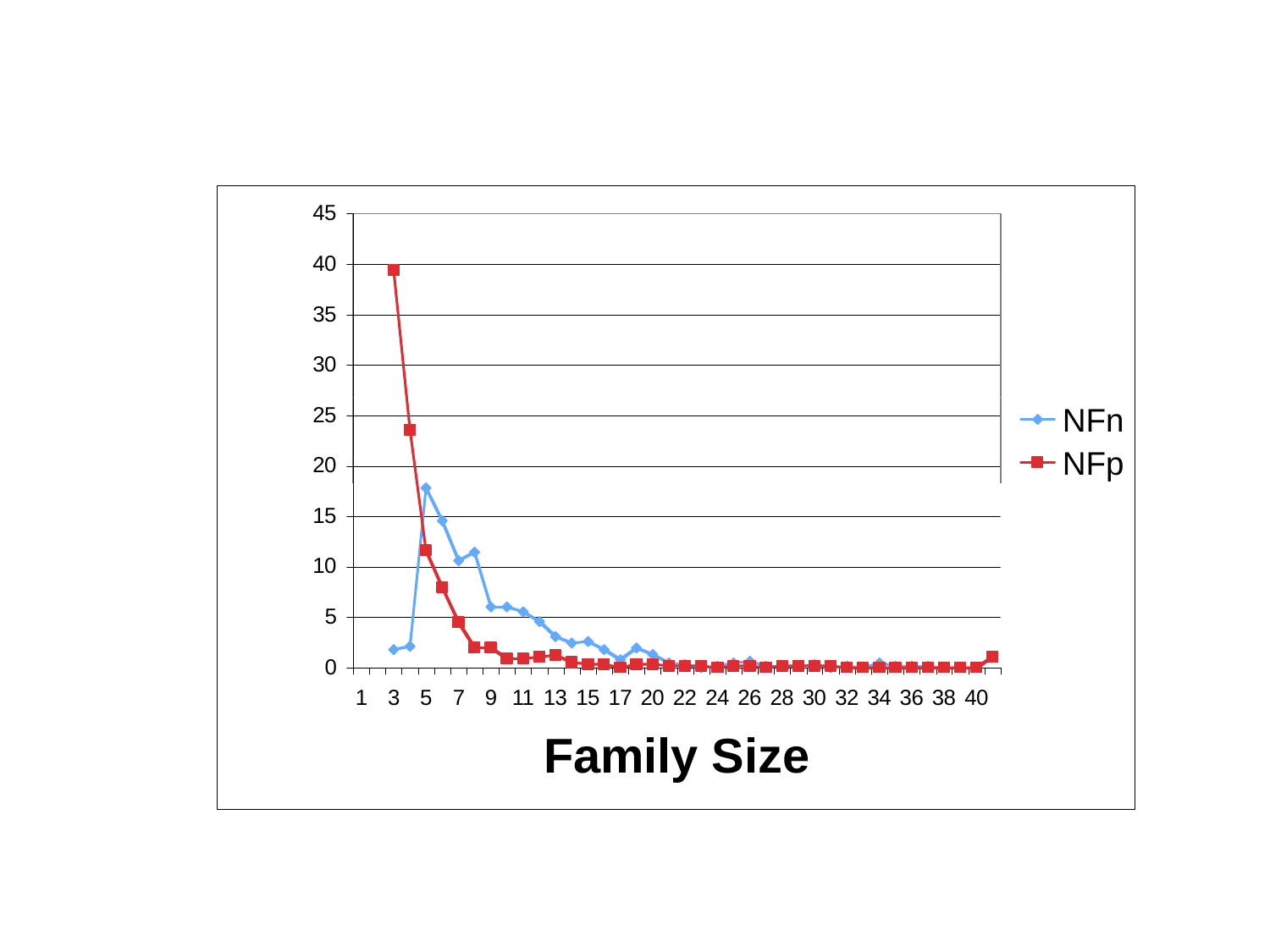

Supplement: Additional file 6 — Size distribution of NFn and NFp groups. This figure shows the different distribution of family sizes by the NFn and NFp groups. [file 1471-2164-10-114-S6.ppt]
